# Bile Peritonitis in Acute Cholecystitis

**DOI:** 10.1155/1990/23517

**Published:** 1990

**Authors:** Roland Andersson, Karl-Göran Tranberg, Stig Bengmark

**Affiliations:** Department of Surgery Lund University S–221 85 Lund Sweden

## Abstract

A review of all patients treated for acute cholecystitis (n=5848) during an 18-year period (1969–1986) at two hospitals (one practising early surgery in patients with acute cholecystitis and the other not) disclosed that 104 (1.8%) had bile within the abdominal cavity at surgery; 71 with a visible perforation of the gallbladder and 33 without. The bile was infected in 82% of performed cultures (most commonly with Escherichia coli). Mortality was 7.7% (8/104 patients), being 20% (4/20)in the hospital practising delayed surgery and 5% (4/84) in the hospital practising early surgery (*p*<0.10). Infectious complications were responsible for the deaths by leading to multiple organ failure with pulmonary or renal insufficiency or gastro-intestinal bleeding. The timing of surgery was the only factor that had prognostic significance, i.e. the longer the hospital delay before surgery the higher the mortality, although elderly patients or patients with perforation tended to have a worse prognosis. In conclusion, the results of this study indicated that early surgery is important in patients with acute cholecystitis as a means of lowering mortality in bile peritonitis in this condition.


*HPB Surgery*, 1990, Vol. 2, pp. 7–13
Reprints available directly from the publisher
Photocopying permitted by license only

© 1990 Harwood Academic Publishers GmbH
Printed in the United Kingdom

### BILE PERITONITIS IN ACUTE CHOLECYSTITIS

ROLAND ANDERSSON, KARL-GÖRAN TRANBERG and STIG BENGMARK

*Department of Surgery, Lund University, S–221 85 Lund, Sweden*

*(Received 16 November 1988)*

A review of all patients treated for acute cholecystitis (n = 5848) during an 18-year period (1969–1986) at two hospitals (one practising early surgery in patients with acute cholecystitis and the other not) disclosed that 104 (1.8%) had bile within the abdominal cavity at surgery; 71 with a visible perforation of the gallbladder and 33 without. The bile was infected in 82% of performed cultures (most commonly with Escherichia coli). Mortality was 7.7% (8/104 patients), being 20% (4/20) in the hospital practising delayed surgery and 5% (4/84) in the hospital practising early surgery ($p < 0.10$). Infectious complications were responsible for the deaths by leading to multiple organ failure with pulmonary or renal insufficiency or gastro-intestinal bleeding. The timing of surgery was the only factor that had prognostic significance, i.e. the longer the hospital delay before surgery the higher the mortality, although elderly patients or patients with perforation tended to have a worse prognosis. In conclusion, the results of this study indicated that early surgery is important in patients with acute cholecystitis as a means of lowering mortality in bile peritonitis in this condition.

KEY WORDS: Bile peritonitis, acute cholecystitis, perforation, surgery, complications.

### INTRODUCTION

The first report on perforation of the gallbladder was published in 1844[1] and has been followed by a number of reports documenting its severity, especially when associated with acute cholecystitis. Morbidity and mortality continues to be high even in fairly recent studies, despite improvements in diagnostic facilities, antibiotics and intensive care[2–4]. This study reviews our experience of bile peritonitis in acute cholecystitis and aims at defining factors of prognostic importance.

### MATERIAL

During the 18-year period 1969 to 1986, 5848 patients were treated with a diagnosis of acute cholecystitis at the Departments of Surgery, Lund University (n = 4940) and Ystad General Hospital (n = 908). This retrospective study was focussed on the 104 patients (1.8% of all patients) that had bile within the abdomen at operation; 71 (68%) patients had a perforated gallbladder, whereas 33 (32%) patients did not have an obvious perforation. In Lund bile peritonitis was found in 84 patients (1.7% of all patients) and in Ystad in 20 patients (2.2% of all patients).

There were 47 women (Lund 38, Ystad 9) and 57 men (Lund 46, Ystad 11) with a mean age of $72 \pm 1$ (SEM) years (Lund $72 \pm 1$, Ystad $75 \pm 3$) and a range of 22 to 94 years. Forty-four (42%) patients had a previous history of gallbladder disease, verified by cholecystography or ultrasonography in 18 patients. Year-by-year distribution of bile peritonitis and relative frequency of perforation were fairly even during the period studied, though there was a tendency towards a lower number of patients with bile peritonitis during the latter half of the study (n = 48) as compared to the first (n = 56).

Treatment policy in patients with acute cholecystitis differed between the two hospitals. In Lund, "emergency" surgery, i.e., with the aim of operating within 24 hr. after admission, was practised between 1980 — 1986, whereas during the previous period (1969–1979), early surgery was advocated, but the timing was less well defined. Diagnostic ultrasonography was introduced during the later half of the study period and was routinely used as an "emergency" diagnostic procedure in patients with clinical signs of acute cholecystitis after 1982.

In Ystad, treatment was more expectant during the whole period studied, aiming at avoiding early surgery during the acute episode of cholecystitis, and operating at a later occasion. Ultrasonography was not available as an emergency investigation.

### *Statistical Methods*

The Mann-Whitney and chi-square tests were used for standard comparison between groups. Analysis of variance with hierarchial classification and analysis of interaction, or, when appropriate, the Mantel-Haenszel test, were used for evaluating the possible influence of various factors on mortality or perforation, while taking into account the variation between the two hospitals. Since mortality and morbidity were almost identical in patients receiving cholecystostomy or cholecystectomy, results from these two procedures were pooled in the following analysis. Values are means ± SEM.

### RESULTS

### *Findings at Admission*

All patients had abdominal pain at admission. In patients with a perforated gallbladder, peritonitis was localized in 83% and generalized in 17%; the corresponding figures for patients without obvious gallbladder perforation was 82 and 18%, respectively. Most (78%) patients had fever (above 38°C), whereas only a few had jaundice (12%) or a palpable mass (8%). The white blood cell count ($13.7 \pm 0.6 \times 10^9/l$; normal $4.0–10.0 \times 10^9/l$) and bilirubin ($33.1 \pm 4.8$ umol/l; normal range 3–20 umol/l) were elevated at admission.

### *Patient's Delay*

Patient's delay was significantly longer in patients with gallbladder perforation than in patients without obvious perforation ($p < 0.05$). Patient's delay was also significantly longer in Ystad than in Lund ($p < 0.05$), and when this was taken into account patient's delay could not be demonstrated to vary with perforation ($p > 0.05$). Fifty-two per cent of patients with gallbladder perforation presented within 24 hours, 28% between 1–3 days and 20% after more than 3 days. The corresponding figures for patients without visible perforation were 70, 21 and 9%, respectively.

### *Hospital Delay*

Seventy patients (67%) were operated upon within 24 hr. after admission. Hospital delay was significantly longer in Ystad (51 ± 10 hours) than in Lund (25 ± 3 hours;

$p < 0.05$). The interval between admission and surgery did not differ between the perforated and non-perforated groups ($p > 0.05$), regardless of whether the difference between the two hospitals was accounted for or not.

### *Diagnostic Procedures*

All 23 ultrasonographic examinations showed signs of cholecystitis, and 9 examinations demonstrated free fluid within the abdominal cavity or localized accumulation of fluid close to the gallbladder. Perforation of the gallbladder was evident in only one of the ultrasound examinations.

The introduction of ultrasonography was associated with a shorter hospital delay (comparison between patients undergoing acute ultrasonography or not in Lund; $p < 0.05$). Twenty of the 23 (87%) patients investigated with ultrasonography were operated within 24 hr. The ratio of perforated/non-perforated gallbladders did not change with the introduction of ultrasonography and more aggressive surgery in patients with acute cholecystitis.

### *Treatment*

Eleven patients received a cholecystostomy during the first two years of the study; otherwise cholecystectomy was performed. Choledocholithotomy was added in 21 patients. All patients received antibiotics, treatment being started before operation in 47% of the patients, during operation in 11% and after operation in 42%.

### *Operative Findings*

At operation, $375 \pm 40$ (mean $\pm$ SEM, range 100–2000) ml bilestained fluid was found within the peritoneal cavity; $485 \pm 55$ (range 100–2000) ml in perforated cases and $175 \pm 20$ (range 100–500) ml in patients without obvious perforation. The abdominal fluid was cultured aerobically and anaerobically in 60 patients and bacterial growth was demonstrated in 49 (82%). Cultures were positive in 87% of patients with and in 73% of patients without perforation ($p > 0.05$). The most commonly isolated microorganism was Escherichia coli (43%), followed by streptococci (10%) and clostridium perfringens (8%).

The perforation was situated in the fundus, corpus and neck of the gallbladder in 45, 40 and 15% of patients, respectively. Acalculous cholecystitis was found in 10 patients, a single stone in 37 and multiple stones in 57. Seventeen patients had an impacted stone in the infundibular area.

Perforation was obvious in 53 (63%) of the 84 patients treated in Lund and in 18 (90%) of the 20 patients treated in Ystad, the difference in perforation rate being statistically significant ($p < 0.05$). Also, perforation was more common in elderly patients than in young patients ($p < 0.05$).

### *Postoperative Complications and Mortality (Tables 1–2)*

Infectious complications dominated the postoperative morbidity and seemed to be responsible for the postoperative deaths by leading to multiple organ failure with pulmonary or renal failure, or gastrointestinal bleeding. The mortality was 20% (4/20) in Ystad and 4.8% (4/84) in Lund, a difference not reaching statistical difference ($p < 0.10$).

**Table 1** Complications

| | *Perforation (n=71)* | *No perforation (n=33)* | *Total (n=104)* |
|---|---|---|---|
| *SURVIVORS* | 64 | 32 | 96 |
| Pulmonary – pneumonia | 5 | — | 5 |
| – atelectasis | 1 | 1 | 2 |
| – pleural effusion | 3 | 1 | 4 |
| – embolius | 1 | — | 1 |
| Wound infection/abscess | 2 | 1 | 3 |
| Urinary infection | 1 | 2 | 3 |
| Cardiac arrest – ventricular fibrillation | 1 | — | 1 |
| Renal failure | 2 | — | 2 |
| Biliary – cutaneous fistula | 1 | 1 | 2 |
| *NON-SURVIVORS* | 7 | 1 | 8 |
| Multiple organ failure | 6 | 1 | 7 |
| Respiratory failure | 5 | 1 | 6 |
| Renal failure | 1 | 1 | 2 |
| Cardiac failure | 3 | 1 | 4 |
| Upper G-I bleeding | 2 | 1 | 3 |

**Table 2** Mortality

| *Year* | *Age (years)* | *Perforation* | *Pat delay* | *Hosp delay* |
|---|---|---|---|---|
| 1. 1969 | 76 | Y | 48 h | 6 h |
| 2. 1972 | 73 | N | 12 h | 3 d |
| 3. 1973 | 91 | Y | 24 h | 2 d |
| 4. 1976 | 84 | Y | 3 d | 2 d |
| 5. 1978 | 66 | Y | 12 h | 2 d |
| 6. 1978 | 70 | Y | 12 h | 3 d |
| 7. 1979 | 89 | Y | 24 h | 24 h |
| 8. 1985 | 91 | Y | 14 d | 3 d |

### *Prognostic Factors*

The risk of postoperative death was larger the longer the interval between admission and surgery ($p<0.01$). However, after eliminating the effect of difference in hospital delay between Lund and Ystad, hospital delay could not be demonstrated to be longer in dying patients ($p>0.05$). No other factor could be demonstrated to vary with prognosis, although perforation and high age tended to be associated with increased mortality. Seven of the eight deaths occurred in patients with a perforated gallbladder; mortality was 9.9% when perforation was obvious and 3.0% when no perforation was found ($p>00.5$). The age of dying patients was $80\pm3$ years as compared to $72\pm11$ years in surviving patients ($p>0.05$). Mortality rate did not vary with patient's delay, physical or laboratory findings at admission or the presence of bacteria in the abdominal cavity (as determined by standard bacterial culture) ($p>0.05$).

As said, "emergency" surgery, usually preceeded by ultrasonagraphy, was practised during the last 7 years of the study period in Lund. At this hospital, mortality was nil during the same period of time.

### DISCUSSION

We conclude from this study that early surgery in acute cholecystitis is important for decreasing mortality in bile peritonitis associated with this condition. Although this conclusion cannot be unequivocally proven by the data obtained, the evidence is quite strong. As far as we know, the management of patients with acute cholecystitis at the two hospitals differed only with respect to the timing of surgery (early vs delayed). If this is true, data from the two hospitals are comparable and can be combined. Under this assumption, it was found that mortality was larger the longer the interval between admission and surgery ($p<0.01$). In addition, the lower relative frequency of perforated gallbladders ($p<0.05$) and the tendency towards decreased mortality ($p<0.10$) in the hospital practising early surgery support the idea that early surgery is important for avoiding death in bile peritonitis. When the effect of the (significant) difference in hospital delay between the two hospitals was eliminated (statistically), hospital delay could not be demonstrated to vary with mortality. This may be interpreted as showing that hospital delay was not an important factor and/or that another factor(s) was a more important determinant of mortality. However, this is unlikely because a/ the basis of the study was the presence of two hospitals in which the treatment was the same except for the timing of surgery, and b/ no other factor could be found to vary with mortality (e.g., patient's delay varied between the hospitals but did not vary with mortality).

In the hospital practising early surgery, the transition from early to emergency surgery in patients with acute cholecystitis, and the increased routine use of emergency ultrasonography, during the last 7 years of the study period was associated with zero mortality in cases of bile peritonitis. Postoperative morbidity and mortality is multifactorial, but it is conceivable that the aggressive surgical attitude contributed to the absent mortality. Also, it appears likely that emergency ultrasonography in patients with suspect acute cholecystitis is helpful in some patients by establishing a definitive diagnosis and by demonstrating signs of imminent or established perforation[5].

Bile peritonitis associated with acute cholecystitis has been reported to have a mortality of 20–40%[2,4,6,7]. In a recent study from Finland, Larmi *et al.* reported that the mortality in acute cholecystitis with free perforation declined from 55% in 1946–1956 to 8% in 1969–1980[3]. They attributed this improvement to a general improvement in pre- and postoperative care, more effective antibiotic therapy and a change from delayed to early (operation within 3–7 days) surgery. However, the importance of the timing of surgery is difficult to determine from their study because they compared two widely different time periods. Our study compared different policies during the same period of time at two hospitals having the same management capacities except for diagnostic ultrasound. A number of other considerations indicates that early rather than delayed cholecystectomy is the method of choice in acute cholecystitis[8,9]. The results of our study support this idea and emphasize the importance of emergency surgery, i.e. surgery within 24 hr.

It should be noted that the incidence of bile peritonitis was not appreciably affected by emergency surgery and that perforation of the gallbladder was equally common at the two hospitals. These findings infer that perforation may occur quite early during the process of cholecystitis and that emergency surgery is advantageous mainly because it shortens the period of free bile in the abdomen. Also, these findings may suggest that intraperitoneal accumulation of bile without obvious perforation is a separate condition. However, it is more likely that it simply represents an earlier stage of the inflammatory process. This interpretation is supported by the finding that the percentage of perforated gallbladders was larger in the hospital with longer patient's and hospital delays.

Unlike other authors[3], we did not find that high age was associated with an increased risk of dying from bile peritonitis. This may, however, be a type II error, and we believe that early surgery in acute cholecystitis is to be recommended also in elderly patients.

### *References*

1. Duncan, J. (1844–45) Femoral hernia; gangrene of the gallbladder; extravasation of bile; peritonitis; death. *North. J. Med.*, **2**, 151–153
2 Felice, P.R., Trowbridge, P.E. and Ferrara, J.J. (1985) Evolving changes in the pathogenesis and treatment of the perforated gallbladder. *Am. J. Surg.*, **49**, 466–473
3. Larmi, T.K.I., Kairaluoma, J.J., Junila, J., Laitinen, S., Ståhlberg, M. and Fock, H.G. (1984) Perforation of the gallbladder. *Acta Chir. Scand.*, **150**, 557–560
4. Riesenfeld, G. (1969) Perforation of the gallbladder. *Int. Surg.*, **52**, 218–225
5. Forsberg, L., Andersson. R., Hederström, E. and Tranberg, K.-G. (1988) Ultrasonography and gallbladder perforation in acute cholecystitis. *Acta Radiologica*, **29**, 203–205
6. Dale, G. and Solheim, K. (1975) Bile peritonitis in acute cholecystitis. *Acta Chir. Scand.*, **141**, 746–748
7. Essenhigh, D.M. (1968) Perforation of the gallbladder. *Br. J. Surg.*, **55**, 175–178
8. Van der Linden, W. and Edlund, G. (1981) Early versus delayed cholecystectomy: the effect of a change in management. *Br. J. Surg.*, **68**, 753–757
9. Norrby, S., Herlin, P., Holmin, T., Sjödahl, R. and Tagesson, C. (1983) Early or delayed cholecystectomy in acute cholecystitis; A clinical trial. *Br. J. Surg.*, **70**, 163–165

*Accepted by S. Bengmark 8 June 1989*

### INVITED COMMENTARY

This paper reviews an uncommon complication of acute cholecystitis — bile peritonitis. The authors report a 1.8% incidence of this complication which was associated with a commendably low 8% postoperative mortality. They have shown that this complication is difficult to diagnose clinically, that the peritoneal bile is usually infected with gram-negative organisms, and that a delay in surgery may increase operative risk especially from infective complications. They conclude that this experience supports a policy of urgent (within 24 hours of admission) surgery for acute cholecystitis.

While early surgery for acute cholecystitis has been shown to be effective and safe in low risk patients[1,2] we must be cautious when drawing conclusions for the overall treatment of a condition on a basis of a retrospective review of a complication, albeit a serious one, which occurs in less than 2% of patients. This is particularly so when a policy of early surgery as practised at Lund has had a disappointing impact on the incidence of bile peritonitis found at operation. Bile peritonitis is not the only cause of death in patients with acute cholecystitis and overall mortality and morbidity must be our prime consideration when deciding management policies.

Although elective cholecystectomy has been reported to be safe in the elderly[3], acute cholecystitis in these patients carries an appreciable mortality much of it attributed to concurrent disease[3,4]. Patients with acute cholecystitis who are at a high surgical risk may be managed non-operatively at first, although early surgery is recommended if they do not improve over 12–24 hours[5]. It would be interesting to know how many patients in this series were treated non-operatively.

*References*

1. Van der Linden, W. and Edlund, G. (1981) Early versus delayed cholecystectomy. The effect of a change in management. *Br. J. Surg.* **68**, 753–757
2. Norrby, S., Herlin, P., Holmin, T., Sjodahl, R. and Tagessan, C. (1983) Early or delayed cholecystectomy in acute cholecystitis? A clinical trial. *Br. J. Surg*, **70**, 163–165
3. Houghton, P.W.J., Jenkinson, L.R. and Donaldson, L.A. (1985) Cholecystectomy in the elderly: a prospective study. *Br. J. Surg.* **72**, 220–222
4. Huber, D.F., Martin, E.W. Jr. and Cooperman, M. (1983) Cholecystectomy in elderly patients. *Am. J. Surgery.* **146**, 719–722
5. Sullivan, D.M., Hood, T.R. and Griffen, W.O. (1982) Biliary tract surgery in the elderly. *Am J. Surg.* **143**, 218–220

L. Blumgart
Inselspital
CH-3010 Bern
Switzerland

